# Supplementary material for: Transcriptomic Analysis Reveals the Inability of Recombinant AAV8 to Activate Human Monocyte-Derived Dendritic Cells
Source: Int J Mol Sci. 2023 Jun 21;24(13):10447. doi: 10.3390/ijms241310447 (PMC10341499; doi:10.3390/ijms241310447)
Supplement: Supplementary file 1 [file ijms-24-10447-s001.zip › ijms-2348562-supplementary.docx]

**Masri, et al, 2023 - Supplementary material**

**Table S1.** Phenotyping of human moDC after differentiation.

| **Donor** | **Phenotype Post-Differentiation** | | | |
| --- | --- | --- | --- | --- |
|  | **% Cells** | **% CD14-** | **% CD14^−^/HLA-DR^+^** | **% CD14^−^/HLA-DR^+^/CD209^+^** |
| **A** | 58 | 99.9 | 99.9 | 94.3 |
| **B** | 41.5 | 97.7 | 99.5 | 65.7 |
| **C** | 78.6 | 99.7 | 99.5 | 96.7 |
| **D** | 50.3 | 99 | 98.9 | 95.2 |
| **E** | 65.3 | 99.8 | 98.8 | 94.4 |
| **F** | 86.5 | 99.8 | 95 | 82.8 |
| **G** | 78.1 | 99.9 | 98.4 | 79.3 |
| **H** | 57 | 98.8 | 99.6 | 99 |
| **I** | 63 | 82.2 | 98.1 | 99.8 |
| **J** | 69.5 | 99.7 | 47.9 | 99.1 |
| **K** | 58.7 | 99.8 | 99.4 | 98.4 |
| **L** | 61 | 99.8 | 99.7 | 94.7 |
| **M** | 41.5 | 99.7 | 99.9 | 98.5 |
| **N** | 66.1 | 99.9 | 99.5 | 99.1 |
| **O** | 55.7 | 99.9 | 99.8 | 98.1 |


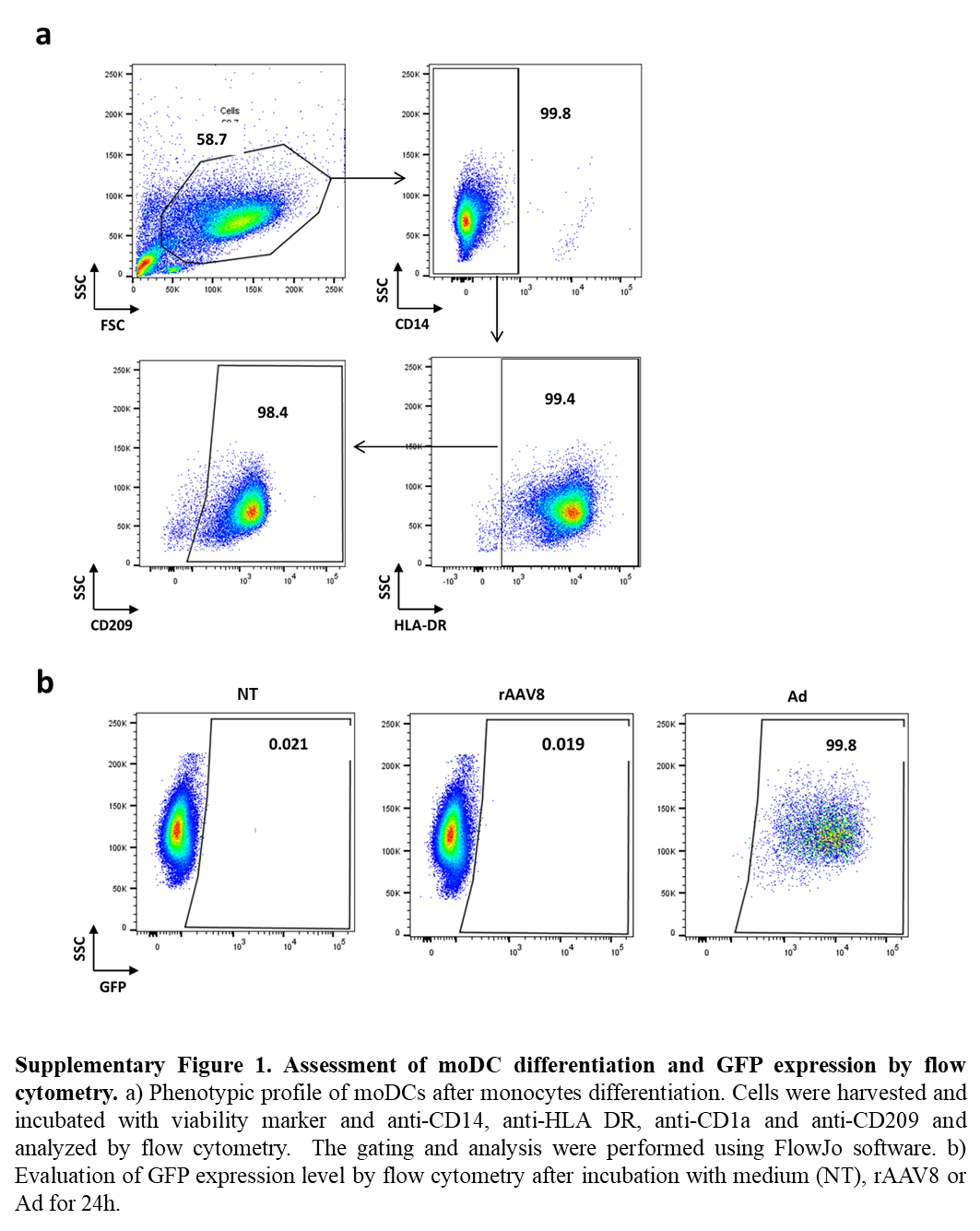


**Supplementary Figure S1.** **Assessment of moDC differentiation and GFP expression by flow cytometry. (a)** Phenotypic profile of moDCs after monocytes differentiation. Cells were harvested and incubated with viability marker and anti-CD14, anti-HLA DR, anti-CD1a and anti-CD209 and analyzed by flow cytometry. The gating and analysis were performed using FlowJo software. **(b)** Evaluation of GFP expression level by flow cytometry after incubation with medium (NT), rAAV8 or Ad for 24 h.


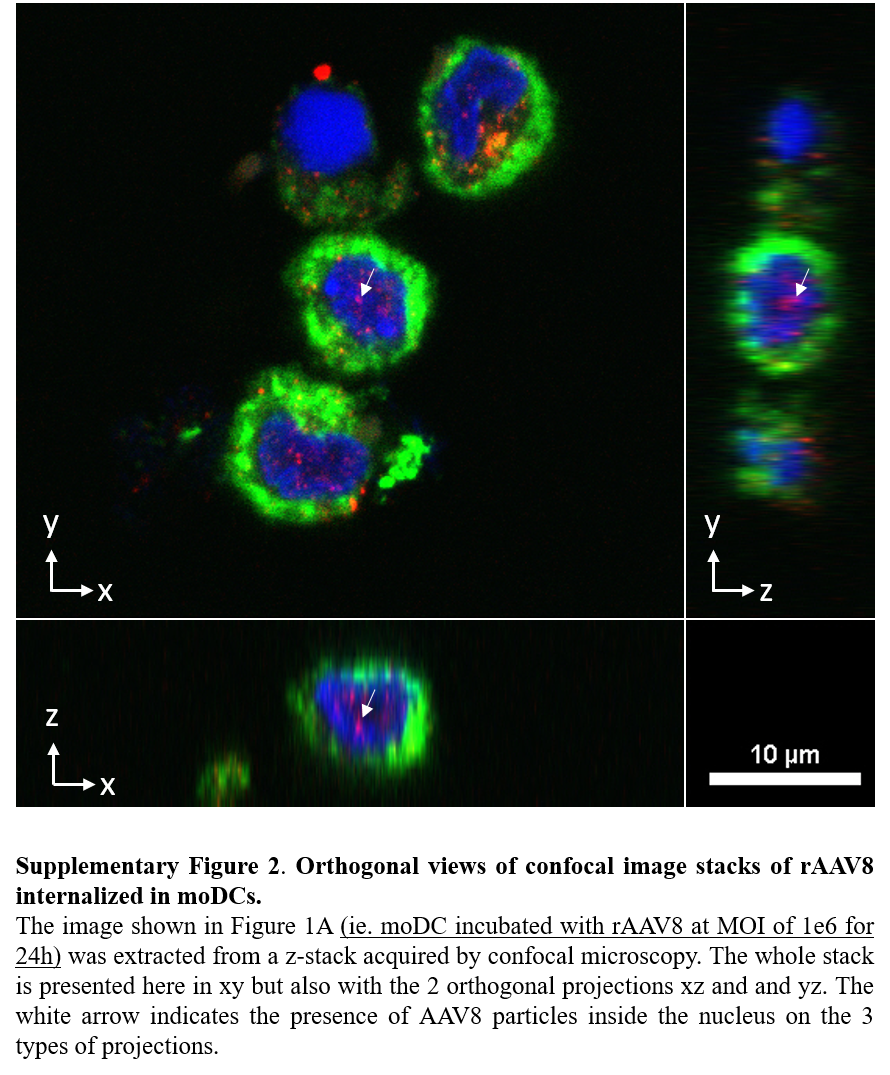


**Supplementary Figure S2.** **Orthogonal views of confocal image stacks of rAAV8 internalized in moDCs.** The image shown in Figure 1A (i.e., moDC incubated with rAAV8 at MOI of 1e6 for 24 h) was extracted from a z-stack acquired by confocal microscopy. The whole stack is presented here in xy but also with the 2 orthogonal projections xz and and yz. The white arrow indicates the presence of AAV8 particles inside the nucleus on the 3 types of projections.


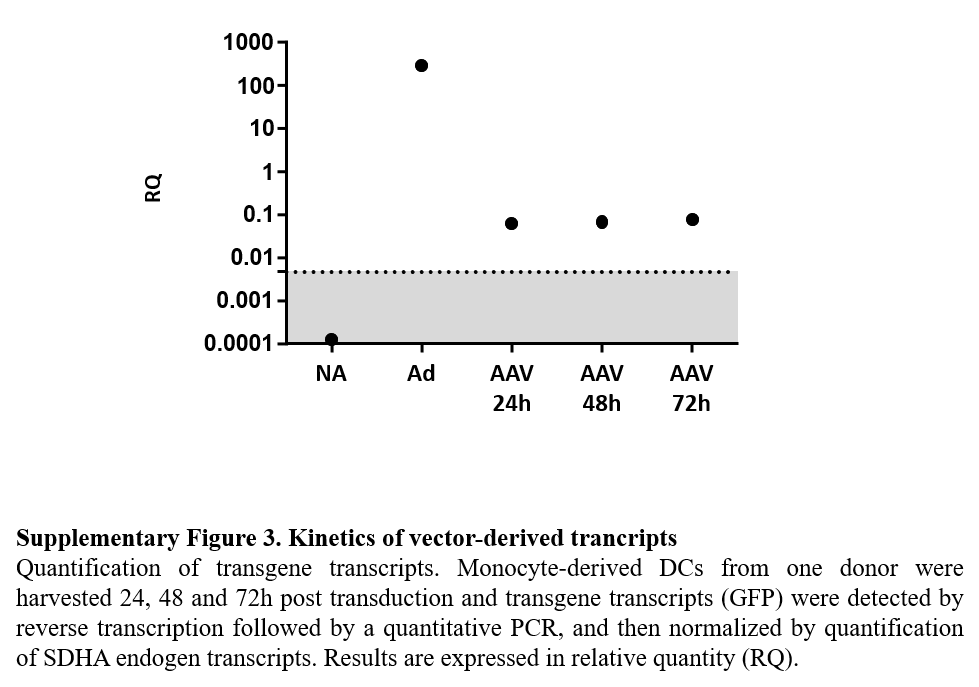


**Supplementary Figure S3. Kinetics of vector-derived trancripts.**

Quantification of transgene transcripts. Monocyte-derived DCs from one donor were harvested 24, 48 and 72 h post transduction and transgene transcripts (GFP) were detected by reverse transcription followed by a quantitative PCR, and then normalized by quantification of SDHA endogen transcripts. Results are expressed in relative quantity (RQ).


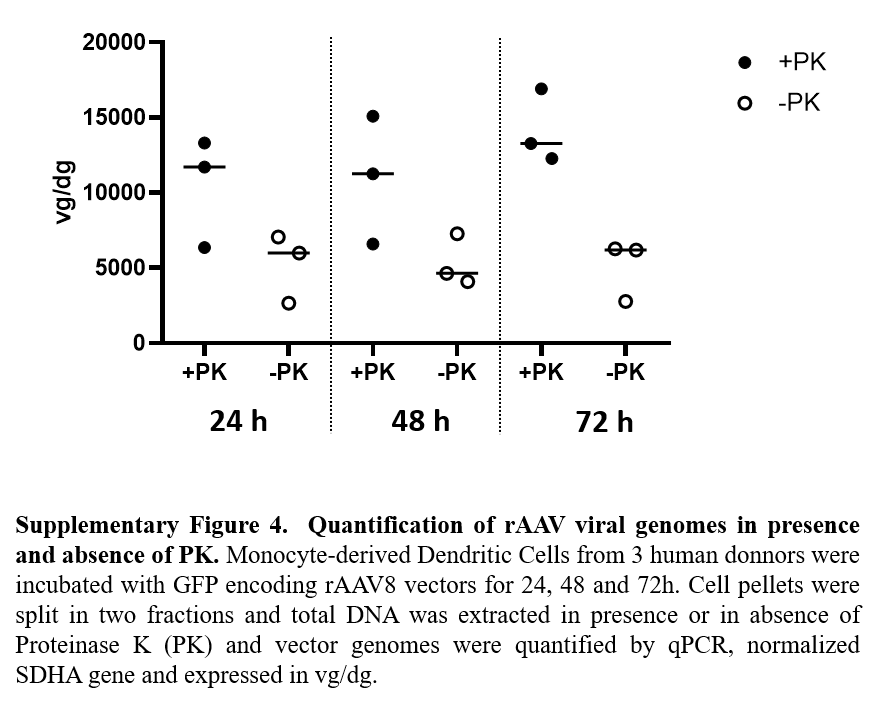


**Supplementary Figure S4.** **Quantification of rAAV viral genomes in presence and absence of PK.** Monocyte-derived Dendritic Cells from 3 human donors were incubated with GFP encoding rAAV8 vectors for 24, 48 and 72 h. Cell pellets were split in two fractions and total DNA was extracted in presence or in absence of Proteinase K (PK) and vector genomes were quantified by qPCR, normalized SDHA gene and expressed in vg/dg.

**Table S2.** Percentage of costimulatory molecules marker expression.

| **Human**  **Donor** | **Costimulatory molecules** | **% of costimulatory molecules marker expression** | | | |
| --- | --- | --- | --- | --- | --- |
|  |  | **Medium** | **LPS/R848** | **Ad** | **AAV8** |
| D | *CD80* | 0.51 | 94.2 | 41.3 | 0.63 |
|  | *CD86* | 0.18 | 94.8 | 32.9 | 0.3 |
| E | *CD80* | 0.12 | NA* | 7.11 | 0.014 |
|  | *CD86* | 0.37 | NA | 28.1 | 1.18 |
| F | *CD80* | 0.093 | 97 | 5.94 | 0.046 |
|  | *CD86* | 0.27 | 53.7 | 57.9 | 1.64 |
| G | *CD80* | 0.18 | 0.088 | 2.9 | 0.29 |
|  | *CD86* | 0.39 | 1.69 | 42.5 | 0.22 |
| H | *CD80* | 0.067 | 81.9 | 17.7 | 0.11 |
|  | *CD86* | 0.44 | 42.8 | 18.4 | 0.099 |
| I | *CD80* | 0.00337 | 68.8 | 14.9 | 3.19 |
|  | *CD86* | 0.24 | 53.2 | 8.46 | 0.34 |
| J | *CD80* | 0.15 | 94.5 | 9.03 | 0.056 |
|  | *CD86* | 0.35 | 68.2 | 27.9 | 0.31 |

* NA: Not Available.


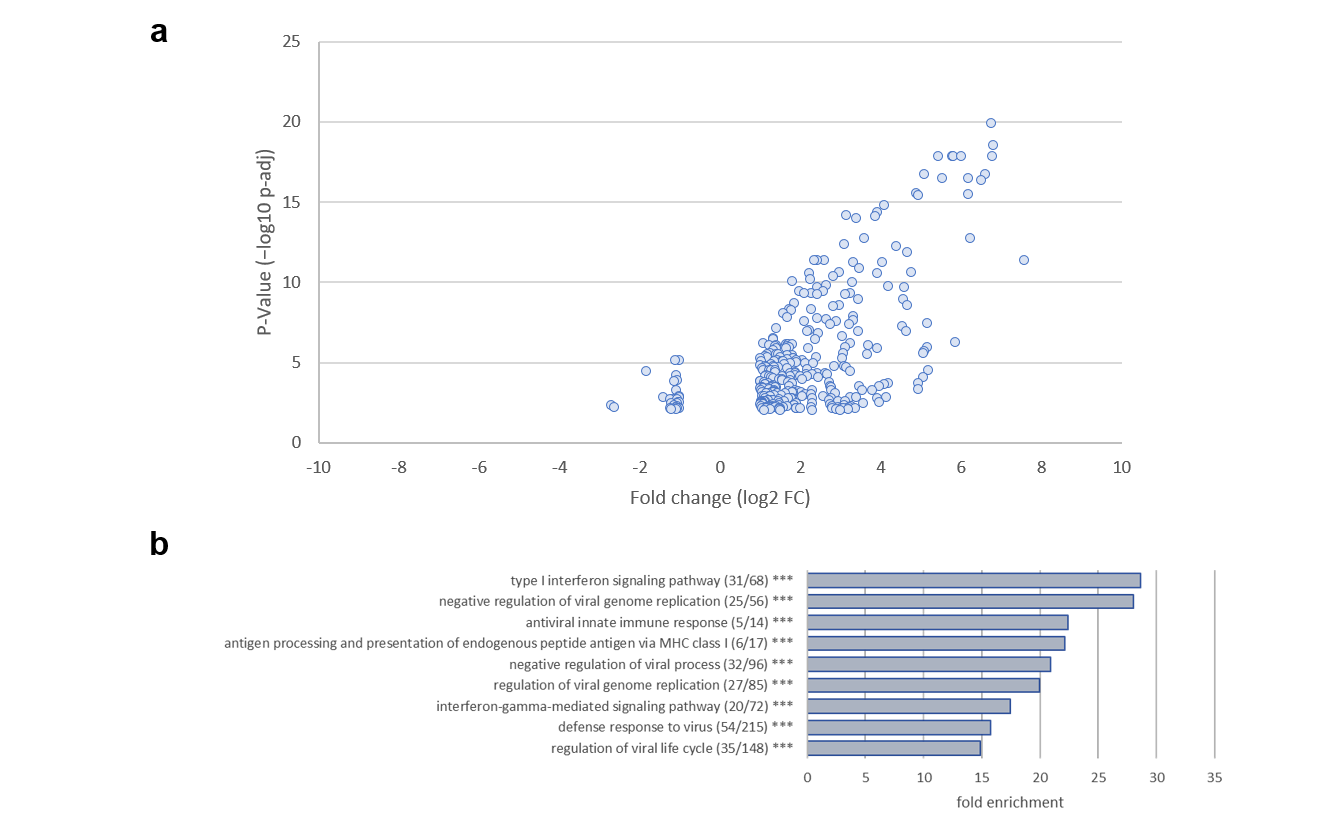


**Supplementary Figure S5. Transcriptomic analysis of adenoviral vector (Ad) effects on human moDCs**. **(a)** Differential expression of genes (DEGs) between NA and Ad (moDCs stimulated by Ad and harvested at 48 h, n = 12). Dots represent DEGs of significantly over expressed genes after Ad stimulation. **(b)** Annotation of DEGs by Enriched GO terms. The 9 most relevant GO terms are presented as histogram. Between brackets are indicated the numbers of annotated genes in our study out of the numbers of genes described in the GO terms. *** *p*-values < 0.001 (Fisher’s exact test with False Discovery Rate *p*-values correction).

**Table S3.** The most 20 differentially expressed genes between the Ad and NA conditions.

| **Gene Name** | **Fold Change** | ***p*-Value** |
| --- | --- | --- |
| *IFI27* | 6.75824707 | 1.36E-20 |
| *APOBEC3A* | 6.80020065 | 3.00E-19 |
| *IFIT2* | 6.7791174 | 1.53E-18 |
| *IFIT3* | 5.77362886 | 1.53E-18 |
| *IFITM1* | 5.42992367 | 1.53E-18 |
| *ISG15* | 5.79628981 | 1.53E-18 |
| *OASL* | 6.00274824 | 1.53E-18 |
| *IFIT1* | 6.59108081 | 1.88E-17 |
| *USP18* | 5.07817826 | 1.88E-17 |
| *MX1* | 5.542916 | 3.49E-17 |
| *NUPR1* | 6.1765115 | 3.49E-17 |
| *RSAD2* | 6.49464506 | 4.62E-17 |
| *IFI6* | 4.89039603 | 3.03E-16 |
| *IFI44L* | 6.1908179 | 3.58E-16 |
| *CCL5* | 4.94354262 | 3.78E-16 |
| *NT5C3A* | 4.09207745 | 1.67E-15 |
| *LY6E* | 3.91832912 | 4.12E-15 |
| *XAF1* | 3.14133096 | 7.08E-15 |
| *HERC5* | 3.87479956 | 8.35E-15 |
| *IRF7* | 3.38173685 | 1.12E-14 |
